# Intermittent Energy Restriction Attenuates the Loss of Fat Free Mass in Resistance Trained Individuals. A Randomized Controlled Trial

**DOI:** 10.3390/jfmk5010019

**Published:** 2020-03-08

**Authors:** Bill I. Campbell, Danielle Aguilar, Lauren M. Colenso-Semple, Kevin Hartke, Abby R. Fleming, Carl D. Fox, Jaymes M. Longstrom, Gavin E. Rogers, David B. Mathas, Vickie Wong, Sarah Ford, John Gorman

**Affiliations:** 1 Exercise Science Program, University of South Florida, Performance and Physique Enhancement Laboratory, Tampa, FL 33620, USA; danielle@prophysique.com (D.A.); lcolensosemple@gmail.com (L.M.C.-S.); kevinhartke@mail.usf.edu (K.H.); arfleming@mail.usf.edu (A.R.F.); cdf0007@auburn.edu (C.D.F.); jlongstrom@mail.usf.edu (J.M.L.); gavin8@mail.usf.edu (G.E.R.); mathasfitness@gmail.com (D.B.M.); vwong@go.olemiss.edu (V.W.); sarahford@mail.usf.edu (S.F.); 2 Team Gorman LLC, Republic, MO 65738, USA; john@team-gorman.net

**Keywords:** fat loss, resistance training, physique enhancement, diet, weight loss, bodybuilding, sports nutrition, refeed, nutrition, diet break

## Abstract

There is a lack of research into how lean, resistance trained (RT) individuals respond to intermittent energy restricted diets. Therefore, we investigated body composition changes in RT-individuals during continuous energy restriction or intermittent restriction. A total of 27 males and females (25 ± 6.1 years; 169 ± 9.4 cm; 80 ± 15.6 kg) were randomized to a ~25% caloric restricted diet Refeed (RF; *n* = 13) or Continuous group (CN; *n* = 14) in conjunction with 4-days/week resistance training for 7-weeks. RF implemented two consecutive days of elevated carbohydrate (CHO) intake, followed by 5-days of caloric restriction each week. CN adhered to a continuous 7-week caloric restriction. Body mass (BM), fat mass (FM), fat-free mass (FFM), dry fat-free mass (dFFM), and resting metabolic rate (RMR) were assessed pre/post-diet. Both groups significantly reduced BM (RF: baseline = 76.4 ± 15.6 kg, post-diet = 73.2 ± 13.8 kg, Δ3.2 kg; CN: baseline = 83.1 ± 15.4 kg, post-diet = 79.5 ± 15 kg, Δ3.6 kg) and FM (RF: baseline = 16.3 ± 4 kg, post-diet = 13.5 ± 3.6 kg, Δ2.8 kg; CN: baseline = 16.7 ± 4.5 kg, post-diet = 14.4 ± 4.9 kg, Δ2.3 kg) with no differences between groups. FFM (RF: baseline = 60.1 ± 13.8 kg, post-diet = 59.7 ± 13.0 kg, 0.4 kg; CN: baseline = 66.4 ± 15.2 kg, post-diet = 65.1 ± 15.2 kg, Δ1.3 kg *p* = 0.006), dFFM (RF: baseline = 18.7 ± 5.0 kg, post-diet = 18.5 ± 4.5 kg, Δ0.2 kg; CN: baseline =21.9 ± 5.7 kg, post-diet = 20.0 ± 5.7 kg, Δ1.9 kg), and RMR (RF: baseline = 1703 ± 294, post-diet = 1665 ± 270, Δ38 kcals; CN: baseline = 1867 ± 342, post-diet = 1789 ± 409, Δ78 kcals) were better maintained in the RF group. A 2-day carbohydrate refeed preserves FFM, dryFFM, and RMR during energy restriction compared to continuous energy restriction in RT-individuals.

## 1. Introduction

The conventional approach for weight loss is adherence to a daily restriction of energy intake to levels that are below weight maintenance requirements for an extended period of time [1]. While this practice is successful for short-term weight loss, the human body responds to chronic energy restriction and weight loss with a range of adaptive metabolic, behavioral, and endocrinological responses that make continued weight loss difficult [1,2,3,4]. A reduced energy expenditure greater than that expected from the reduction in body mass [5] is one of the most studied aspects of the body’s adaptive responses to continued weight loss. This “adaptive thermogenesis” contributes to weight loss plateaus and weight regain after cessation of an energy-restricted diet.

‘Non-Linear Dieting’ (also known as intermittent energy restriction) can be utilized as a strategy to attenuate this adaptive response, thereby increasing weight loss during subsequent periods of energy restriction [1]. Non-linear dieting involves intermittent periods of energy balance throughout the diet, as opposed to continuous long-term energy restriction. Non-linear dieting can be incorporated in various strategies of cycling caloric intake over daily and/or weekly time periods, including total fasting on alternate days [6], intermittent fasting [7], time-restricted feeding [8], and diet breaks [9].

The ability of each of these non-linear dieting methods to attenuate adaptive responses to energy restriction are varied, with some investigations reporting favorable outcomes and others reporting no definitive benefits when compared to continuous energy restricted diets. An alternate day total fasting study produced similar changes in body composition and resting metabolic rate as compared to a moderate calorie-restricted diet in obese participants over an 8-week period [10]. Additionally, when obese subjects were instructed to consume 100% of their caloric intake during an 8-h period each day (a time restricted feeding investigation), it produced mild caloric restriction and weight loss outcomes when compared to a non-dieting control group [8]. “Diet breaks” are typically characterized by taking short, defined periods of time off from a diet—e.g., a 2-week break. When researchers instructed obese males to cycle between reduced energy intakes (33% reduction) and maintenance calories for 2-week blocks, greater weight and fat loss was achieved when compared with continuous energy restriction. The authors suggested that such diet breaks may reduce compensatory metabolic responses and, in turn, improve weight loss efficiency [9].

The majority of the research on adaptive thermogenesis and subsequent non-linear dieting approaches has utilized overweight and obese populations. There is a dearth of scientific inquiry into healthy, non-overweight individuals, who may be motivated to lose weight to improve physical appearance and enhance self-perception as opposed to health outcomes [11]. For this population, weight loss efforts aim to reduce fat mass while maintaining/gaining fat-free mass by implementing a caloric deficit with a relatively high dietary protein intake, a slow rate of weight loss, and engaging in a resistance training program [12,13,14,15].

The adaptive responses resulting from energy restriction which oppose ongoing weight loss efforts are similar in lean and obese individuals [4]. It is not uncommon for recreational and competitive physique athletes to incorporate a planned, periodic refeeding during their weight loss programs [3,16,17]. In a narrative review focused on metabolic adaptations for the athlete, Trexler et al. [3] described a refeed as consisting of a brief overfeeding period in which caloric intake is increased above the current energy-restricted diet levels (often to maintenance or slightly above maintenance levels). The increase in caloric intake is predominately achieved by increasing carbohydrate consumption. These carbohydrate-based refeeds are typically 1–3 days in duration and are implemented on a weekly basis. Trexler and colleagues further described the proposed goal of periodic refeeds as a temporary increase in leptin and a stimulation of metabolic rate [3]. It is also possible that incorporating intermittent carbohydrate-based refeeds during an energy-restricted diet in resistance trained individuals may help preserve fat-free mass by allowing for a greater volume and intensity of resistance training due to the concomitant elevations in skeletal muscle glycogen.

The primary purpose of this study was to compare body composition changes in resistance trained individuals after 7 weeks of either continuous energy restriction or intermittent restriction with a 2 days per week of carbohydrate refeeding during a supervised daily undulating resistance training program. A secondary purpose of the current investigation was to observe the adaptive responses to the energy-restricted diet via the reductions in resting metabolic rate and leptin concentrations. Our hypothesis is that regardless of diet assignment (continuous energy restriction or intermittent restriction, muscle mass and resting metabolic rate will be maintained during the dietary intervention due to the inclusion of a resistance exercise stimulus and a relatively high dietary protein intake. Due to a lack of existing research for including intermittent energy restriction in a resistance-trained lean population, we were unsure if positive body composition and resting metabolic rate outcomes would be observed. To the best of our knowledge, this is the first dietary intervention to investigate a carbohydrate-based refeed in resistance trained individuals seeking to optimize their physiques.

## 2. Materials and Methods

### 2.1. Study Design

This study utilized a parallel-groups, repeated-measures design wherein participants were randomized to a reduced calorie diet or an isocaloric non-linear diet that included a 2-day carbohydrate refeed (5 days of a reduced calorie diet and 2 days of non-dieting maintenance caloric intake) in conjunction with a supervised resistance training program for 7 weeks. Participants visited the laboratory on three occasions. The first visit included screening, familiarization to the study procedures, and the initiation of tracking all food intake for the next 2 weeks to determine maintenance calories (the habitual caloric intake of each participant such that they neither gained nor lost body mass). The final two laboratory visits took place immediately prior to and after the 7-week diet and supervised resistance training program. The primary dependent variable (DV) was body composition (fat-free mass (FFM), dry fat-free mass (dFFM), fat mass, and body fat percentage). A secondary DV included resting metabolic rate (RMR). Additionally, fasting leptin levels were measured in a subsample of participants for descriptive purposes only.

### 2.2. Participants

Using data from a similar population in which lean athletes were under investigation for weight loss [12], the effect size calculated for a decrease in fat mass over a period of several months (using a caloric restriction of 20%) was 0.5. Using this calculated effect size, an alpha level of 0.05, and a power of 0.95, a sample size of approximately 12 subjects per group was estimated (not considering attrition rates). Fifty-eight healthy, resistance trained males and females were recruited for participation. Four of these participants never completed baseline testing and an additional three participants never initiated the resistance training workout. An additional 21 participants withdrew during the study on their own volition. Three participants completed all aspects of the study but were withdrawn before data analysis due to self-admitted non-compliance with the dietary protocol. Twenty-seven participants completed all aspects of the diet intervention and were included in the final data analysis. Table 1 provides participant demographics. Inclusion criteria required resistance training experience (self-attested by the participants). Informed consent was obtained by all participants and the study was approved by the University of South Florida Institutional Review Board (Pro00030602) and is in compliance with the Declaration of Helsinki as revised in 1983. There were no significant differences between groups at pretraining for any demographic or dependent variable of interest.

### 2.3. Diet and Exercise Intervention

**Figure 1:**
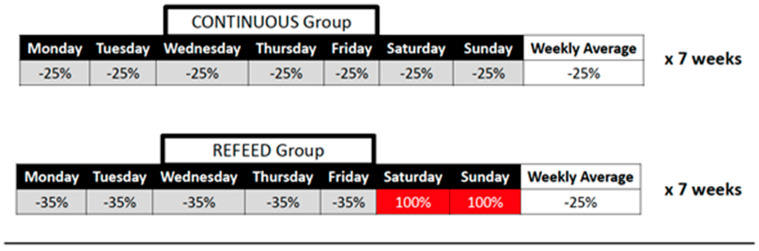
Caloric restriction during a typical week of dieting for both isocaloric diet treatments.

Participants were randomly assigned to a non-linear diet (refeed) or continuous diet group. Prior to baseline testing, participants tracked their typical diets for 2 weeks to determine maintenance caloric intake. All participants were placed on a diet that prescribed a 25% reduction from maintenance calories with a dietary protein intake of 1.8 g of protein/kg body mass and remaining calories split evenly between fat and carbohydrate. The continuous group adhered to a daily 25% caloric deficit for 7 weeks. The refeed group reduced caloric intake by 35% for 5 days (typically Monday–Friday) and consumed the predetermined maintenance calories for two consecutive days (typically Saturday–Sunday). Participants were instructed to comprise their increased “refeed” calories in the form of carbohydrate only. Figure 1 provides an overview of the two different diet configurations. Participants were assigned a personal nutrition coach to answer any diet-related questions throughout the study. Additionally, participants consumed whey protein isolate after each resistance exercise session (25 g of ISO-100 from Dymatize Nutrition).

The resistance training program consisted of two upper-body focused days and two lower-body focused days per week during weeks 1–3 and 5–7, and two workouts during week 4 of the training program. The reduced training frequency during week 4 was a preplanned reduction in volume and served as a taper. Upper-body workouts consisted of six exercises per session and required that the participants complete chest press, barbell rows, overhead press, and assisted pull-ups (or lat pulldown) each week and then choose from a list of other upper body exercises to complete the required number of exercises for the workouts. The set and repetition ranges varied throughout the program, including three initial sets and progressing to four sets of 6–8 and 8–10 repetitions. The lower-body workouts consisted of five exercises per session and required that each participant complete deadlifts and hip thrusts each week for two of the exercises and then choose from a list of other lower body exercises to complete the required number of exercises for the workouts. Lower body progressions followed a similar structure as upper body, with three initial sets and progressing to four sets of 6–8 and 8–10 repetitions (with the exception of calf raises, which were competed with 12–15 repetitions per set for the entire 7-week program). A sample lower-body (Table S1) and upper-body (Table S2) workout from the final week of the study (week 7) are included as supplemental files.

Participants self-selected the load that would allow them to complete the appropriate number of repetitions within the specified range, terminating each set approximately one repetition from failure. Each workout was supervised by research assistants. Additionally, participants were instructed to perform 30-min of aerobic exercise (running, cycling, swimming, rowing, etc.) at a low to moderate intensity (at a pace in which they could have a conversation with a running partner) twice per week.

### 2.4. Baseline and Post-Intervention Testing

Pre-diet baseline testing was scheduled following the 2 weeks of diet tracking to determine maintenance calories. Participants were instructed to fast overnight and refrain from physical activity for 48 h prior to testing. After the 7-week diet period ended, a post-intervention assessment was scheduled that was identical to the pre-diet baseline testing assessment. In order to control for any increase in glycogen storage and the retention of water that is associated with increased glycogen stores, the post-diet session was scheduled for at least 2 days following the last refeed day for those subjects randomly assigned to the refeed group.

### 2.5. Total Body Water Assessment

Upon entering the laboratory, participants urinated and then had their body height measured on a physician beam scale (Health-O-Meter; model 402KL; Pelstar, Inc., McCook, IL, USA). Next, body weight and total body water was measured with multi-frequency bioelectrical impedance analysis. Multi-frequency bioelectrical impedance analysis was completed with an InBody^®^ 570 Body Composition Analyzer (Biospace, Inc. Seoul, Korea). The electrodes are situated beneath the participant’s feet on the platform and on the palms and thumbs attached to handles of the unit. Total body water, segmental impedance, extracellular and intracellular water were all measured with the subject’s bare skin in contact with the electrodes. Body mass and impedance are automatically assessed through the manufacturer software. After the device obtained subject weight the participant was then instructed to stand erect, arms extended and not touching the side of the body. Due to the nature of the investigational procedures in relation to having participants in the refeed treatment increase carbohydrate intake and the potential for increased water retention, total body water was measured and utilized to estimate dry fat free mass. Dry fat free mass was calculated as fat free mass minus total body water.

### 2.6. Resting Metabolic Rate (RMR) Assessment

RMR was assessed using a Parvo Medics’ TrueOne^®^ 2400 (ParvoMedics, Sandy, UT) integrated metabolic measurement system. The metabolic measurement system was calibrated prior to every assessment. RMR was assessed for 20 min as participants lay supine under a hood. The first 5 min were discarded and the remaining time of the test was averaged [18] for the calculation of RMR. Due to a mechanical issue with the primary metabolic measurement system, seven (five from the continuous group and two from the refeed group) subjects’ RMR was assessed at baseline and post-intervention using a Cosmed FitMate Pro™ (Cosmed, Italy). Similar to the primary metabolic measurement system, the first 5 min of RMR data collection was discarded.

### 2.7. Body Composition Assessment

Body composition was assessed using the BodyMetrix™ BX-2000 A-mode ultrasound (IntelaMetrix, Livermore, CA, USA) with a standard 2.5 MHz probe according to procedures as previously described [14]. All body composition assessments were completed by the same two trained technicians. The calculated bodyfat % test–retest reliability was intraclass correlation = 0.99; SEM = 0.196% fat; minimal detectable difference = 0.54% fat (Technician #1) and intraclass correlation = 0.99; SEM = 0.58% fat; minimal detectable difference = 1.6% fat (Technician #2). Each technician performed the body composition on the same participants at baseline and post-intervention. We were not able to obtain ultrasound body composition measures for one participant. In this instance, the body composition data was obtained from the multi-frequency bioelectrical impedance analysis (InBody^®^ 570 Body Composition Analyzer; Biospace, Inc., Seoul, Korea).

### 2.8. Fasting Leptin Assessment

A subsample of participants (*n* = 8; seven males and one female) provided fasting blood samples to assess plasma leptin via venipuncture from an antecubital vein in the forearm according to standard phlebotomy procedures. The plasma leptin test was developed and its analytical performance characteristics were determined by Quest Diagnostics Nichols Institute (San Juan Capistrano, CA, USA) via radioimmunoassay.

### 2.9. Statistical Analysis

Descriptive statistics (mean ± SD) for all dependent variables were calculated. An independent samples *t*-test was conducted to assess baseline differences between groups. Data for all DVs were analyzed by a two group (refeed diet vs. continuous diet) × two time (pre-diet and post-diet) between–within factorial analysis of variance with repeated measures on the second factor via a per protocol analysis. Any missing weekly nutrition data was replaced with the average dietary intake for that variable from the other weeks of submitted dietary tracking for that participant. For each outcome, an effect size was calculated as the pretest–posttest change, divided by the pooled pretest SD. For any main effects observed, a post-hoc paired samples *t*-test was conducted. All analyses were completed using SPSS (version 22; IBM, Armonk, NY, USA) software, and the alpha criterion for significance was set at 0.05.

## 3. Results

Dietary intake data are summarized in Table 2. There were no significant differences between the two diet groups for any dietary intake variable. The caloric restriction was 26% and 21% for the refeed and continuous diet groups, respectively.

**Figure 2:**
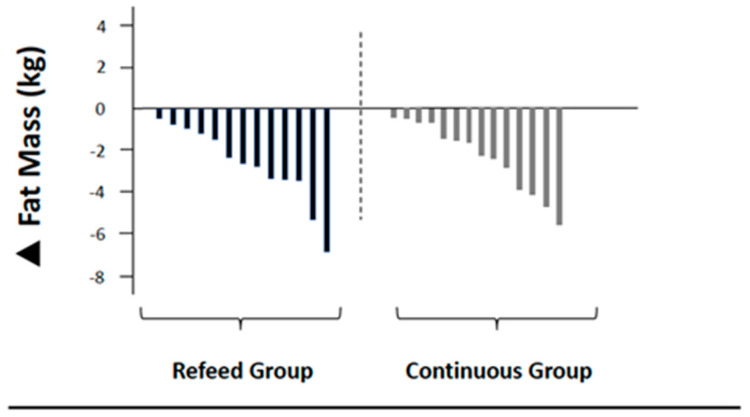
Individual participant changes in fat mass.

**Figure 3:**
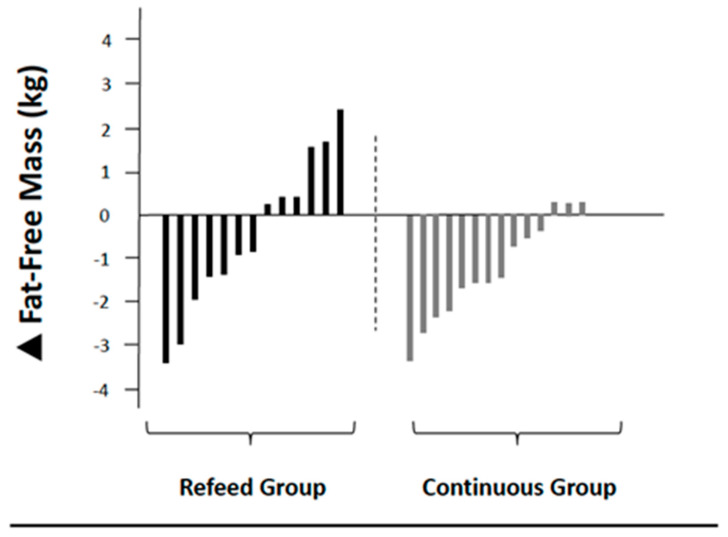
Individual participant changes in fat-free mass.

Body composition and RMR data are summarized in Table 3. There were no significant differences at baseline between groups. Figure 2 and Figure 3 display the individual participant changes in fat mass and fat-free mass. The skewness and kurtosis coefficients were within a range of ±1.60 and a visual inspection of their histograms, normal Q-Q plots, and box plots demonstrated that the data were normally distributed [19].

There were no significant differences between groups for total workouts completed, upper body training volume, lower body training volume, or total body training volume. Adherence to the workout program was satisfactory with participants completing an average of 24 of the 26 scheduled workouts (>90% adherence). Plasma leptin levels significantly declined from 3.9 ± 1.1 to 1.6 ± 0.67 ng/mL in a pooled subsample of eight participants during the 7-week dietary period. Raw data for each participant is available as supplemental file 1 (Table S3).

## 4. Discussion

To the authors’ knowledge, this is the first study to assess the effects of a 2-day carbohydrate refeed to counter metabolic adaptations associated with caloric restriction in a lean, resistance trained population. The primary finding of this study is that a consecutive 2-day carbohydrate refeed preserves fat-free mass/dry fat-free mass during an energy-restricted diet as compared to continuous energy restriction in a population that prioritizes the maintenance of muscle mass when dieting. A secondary finding was that resting metabolic rate was better maintained, albeit slightly, with the 2-day carbohydrate refeed. In a subsample of participants, plasma leptin levels were predictably decreased during the weight loss period.

For individuals seeking to optimize their physiques, maintaining muscle mass while concomitantly reducing fat mass is of utmost importance. While energy deficits are associated with a decline in body weight, the loss of body weight is not solely comprised of body fat stores, but also typically includes the loss of skeletal muscle mass [20,21]. In lean, physically active males and females (a similar participant population as the current study), Pasiakos and colleagues [22] reported that a 10-day diet (≈20% energy restriction) resulted in a 19% decrease in skeletal muscle protein synthesis rates. In another study investigating lean, physically active males and females, it was reported that muscle protein breakdown was elevated by 60% in response to a 10-day, 20% energy deficit [23]. Taken together, these studies demonstrate a net catabolic effect in response to moderate energy restriction in lean individuals [22,23]. Indeed, during contest preparation phases (which typically involve extended periods of energy restriction), competitive physique athletes experience losses of fat mass and fat-free mass [24,25,26]. In the current investigation, several efforts were made to prioritize the maintenance of fat-free mass during energy-restriction. These efforts included a slow rate of weight loss, a relatively high protein intake of 1.8 g/kg body mass per day, and the inclusion of a resistance exercise program. In lean individuals, evidence exists that faster rates of weight loss [12], low protein intakes [13,27], and the absence of a resistance exercise stimulus [28,29,30] when dieting contribute to a greater proportion of fat-free mass losses relative to the total weight loss achieved.

There are a few potential explanations as to how the refeed diet may have prevented the loss of fat-free mass. Simply not being in a caloric deficit for 2 days per week (or being in a caloric deficit for no more than five consecutive days) could have blunted the catabolic environment for skeletal muscle as demonstrated when dieting for 10 consecutive days [22,23]. It could also be hypothesized that the twice-weekly increase in carbohydrate intake elevated skeletal muscle glycogen stores. Such increases may have resulted in less fatigue and subsequently greater effort during the workouts following the carbohydrate refeeds. Unfortunately, there were no assessments of skeletal muscle glycogen during the intervention, so proposed elevations can only be speculative. In addition, the total lifting volume was not different between the two groups.

Elevated carbohydrate intakes increase endogenous insulin concentrations. Carbohydrate intake and the subsequent insulin secretion results in the suppression of acute skeletal muscle protein breakdown in diverse populations, including lean resistance trained individuals [31,32,33]. While it is possible that the 2-day carbohydrate refeeds increased insulin levels which may have suppressed skeletal muscle protein breakdown leading to a greater preservation of fat-free mass, insulin levels were not measured throughout the investigation and therefore such reasoning is speculative at this time.

In the current investigation, the 2-day carbohydrate refeeds reduced the suppression of RMR to a greater extent than did the continuous dieting group. The maintenance of fat-free mass in the refeed group is the likely explanation for the preservation of RMR, as approximately 75% of the variability of RMR is predicted by fat-free mass [34]. Weight loss consistently leads to unfavorable reductions in RMR, and is especially potent in lean individuals [17,25,26,35]. In a previous investigation utilizing lean females, it was reported that a 3-day carbohydrate overfeeding period did not alter resting metabolic rate [36]. Given this finding, we are led to believe that the protection of RMR was better explained by the maintenance of fat-free mass as opposed to the 2-day carbohydrate refeeding employed. However, it is possible that the 2-day carbohydrate refeed was responsible for the maintenance of the fat-free mass. If so, then there was an indirect benefit of the 2-day carbohydrate refeed on the maintenance of the RMR observed in those assigned to the refeed treatment.

The decrease in leptin concentrations observed in a subsample of our participants is consistent with previous findings in lean populations. Decreases in leptin concentrations have been reported in several case studies of male [25] and female [37] physique athletes. Unfortunately, with only a subsample of participants providing leptin samples we were not able to determine if there were any differences between the refeed and continuous energy deficit treatments. Carbohydrate overfeeding has been shown to positively influence fasting leptin levels. In a group of lean females, 3-day carbohydrate overfeeding (40% excess energy) led to a 28% increase in plasma leptin concentration [36]. There were two main differences in this investigation as compared to the current study. First, the participants in the Dirlewanger et al. [36] study were not increasing energy intake back to maintenance levels from a caloric deficit (as was done in our investigation), but rather were overfeeding carbohydrate above weight maintenance levels. Second, plasma leptin concentrations were measured the morning immediately after the last day of the carbohydrate overfeed. In our investigation, fasting leptin levels were assessed no sooner than 2 days after the last day of the carbohydrate refeed, and therefore the leptin concentrations were more reflective of a chronic weight loss effect and not in response to a carbohydrate refeed [38].

Some have postulated that achieving energy balance (or the absence of energy restriction) appears to be important in order to attenuate the effects of the adaptive responses to energy restriction [1,39]. A number of investigations have reported that participants consume less than their weight maintenance energy requirements during refeeding days, and subsequently are in varying degrees of energy restriction for the duration of the intervention [1,6,40,41]. In a comprehensive review on this topic, Sainsbury and colleagues [1] summarized all known randomized controlled trials in overweight/obese adults in which energy balance was intermittently restored. Of these five investigations, two resulted in greater weight loss in the intermittent energy restriction than in the continuous energy restriction [9,42] and the three others reported that weight loss was comparable between intermittent energy restriction and continuous energy restriction [43,44,45]. In the current study, the participants assigned to the 2-day carbohydrate refeed intervention met the condition of achieving energy balance during refeed days as their caloric intakes averaged approximately 19 calories above pre-diet weight maintenance energy requirements.

There were several inherent limitations in the current investigation. Resting metabolic rate was measured using a different device in a small portion of the subjects due to an equipment malfunction in our primary device. However, all participants were assessed on the same device for baseline and post-diet testing. Not measuring acute insulin changes during the 2-day carbohydrate refeeds limited our ability to describe potential mechanisms for the preservation of fat free mass that was observed. Despite these shortcomings, our study had several notable strengths, including the employment of supervised workouts and personalized nutritional counseling. Additionally, we took preventative steps to ensure that the fat-free mass measures were not biased by the elevated carbohydrate intake by requiring all post-diet assessments be at least 2 days after the carbohydrate refeed and by accounting for the total body water content of each participant. The practical recommendations of the study are such that if a lean, resistance-trained individual seeks to reduce caloric intake for the purpose of fat loss, this should be undertaken with relatively high protein intakes, a slow rate of weight loss, and periodic carbohydrate refeeding. Such considerations appear to support the maintenance of fat-free mass and resting metabolic rate during caloric restriction.

## 5. Conclusions

In summary, this is the first investigation, to our knowledge, to demonstrate a preservation of fat-free mass and resting metabolic rate in response to a 2-day carbohydrate refeed during an energy restricted diet in lean, resistance trained males and females. The attenuation of these adverse responses to caloric restriction may have been dependent on the restoration of true energy balance (or the interruption of continued energy restriction) in the carbohydrate refeed group. Our findings suggest that, in lean individuals, the inclusion of resistance training, high protein intakes, a slow rate of weight loss, and periodic carbohydrate refeeding may prevent some of the adverse responses to prolonged energy restriction. Future work in this area should determine the effects of single intermittent refeed days (i.e., every third day) as opposed to two consecutive days.

## Figures and Tables

**Table 1:** Characteristics of participants at baseline (mean ± SD).

Variable	Refeed	Continuous
Male (#)	*n* = 6	*n* = 8
Female (#)	*n* = 7	*n* = 6
Age (yrs)	26.3 ± 8.0	24.2 ± 3.7
Height (cm)	166.4 ± 9.9	172.2 ± 8.2
Body Mass (kg)	76.4 ± 15.6	83.1 ± 15.4
RT Experience (yrs)	4.9 ± 6.0	5.1 ± 4.7

# = Number of male and female participants within each treatment. RT = resistance training.

**Table 2:** Macronutrient intake (mean ± SD).

	Refeed (*n* = 13)		Continuous (*n* = 14)	
Nutrient Variable	Baseline	Diet	Baseline	Diet
Kcals	2012 ± 452	1488 ± 371^↓^	2042 ± 452	1617 ± 402^↓^
CHO (grams)	217 ± 55	123 ± 52^↓^	217 ± 51	132 ± 37^↓^
PRO (grams)	109 ± 34	130 ± 23^↑^	109 ± 32	140 ± 26^↑^
Fat (grams)	79 ± 21	53 ± 15^↓^	82 ± 22	59 ± 20^↓^
Kcal/kg body mass	27 ± 5.5	20 ± 4.6^↓^	25 ± 4.0	19 ± 3.5^↓^
CHO (g/kg/day)	2.9 ± 0.7	1.7 ± 0.6^↓^	2.6 ± 0.5	1.6 ± 0.4^↓^
PRO (g/kg/day)	1.4 ± 0.4	1.8 ± 0.1^↑^	1.3 ± 0.3	1.7 ± 0.2^↑^
Fat (g/kg/day)	1.1 ± 0.3	0.7 ± 0.2^↓^	1.0 ± 0.2	0.7 ± 0.2^↓^
CHO/PRO/Fat (%)	43-22-35	33-35-32	43-21-36	33-34-33

CHO = carbohydrate; PRO = protein; g/kg/day = grams/kilogram body mass/day. ↓Significant decrease from baseline (*p* < 0.05). ↑Significant increase from baseline (*p* < 0.05).

**Table 3:** Body composition and resting metabolic rate (mean ± SD).

	Refeed				Continuous			
Variable	Pre	Post	Change (CI)	ES	Pre	Post	Change (CI)	ES
Body weight (kg)	76.4 ± 15.6	73.2 ± 13.8 *	−3.2 (−1.4; −5.1)	0.22	83.1 ± 15.4	79.5 ± 15.0 ^#^	−3.6 (−2.6; −4.6)	0.24
Fat mass (kg)	16.3 ± 4.0	13.5 ± 3.6 ^#^	−2.8 (−1.7; −3.9)	0.74	16.7 ± 4.5	14.4 ± 4.9 ^#^	−2.3 (−1.4; −3.3)	0.49
Body fat (%)	21.6 ± 4.6	18.8 ± 6.8 ^#^	−2.8 (−1.9; −3.8)	0.49	20.6 ± 6.1	18.6 ± 6.8 ^#^	−2.0 (−1.1; −3.0)	0.31
FFM (kg)	60.1 ± 13.8	59.7 ± 13.0	−0.4 (−1.5; 0.6)	0.03	66.4 ± 15.2	65.1 ± 15.2 *	−1.3 (−1.9; −0.6)	0.09
Dry FFM (kg)	18.7 ± 5.0	18.5 ± 4.5 ^∧^	−0.2 (−0.7; 0.3)	0.04	21.9 ± 5.7	20.0 ± 5.7 ^∧^	−1.9 (−2.7; −1.2)	0.33
RMR (kcals)	1703 ± 294	1665 ± 270	−38 (−141; 62)	0.13	1867 ± 342	1789 ± 409 *	−78 (−139; −16)	0.21

Note. FFM = fat-free mass; RMR = resting metabolic rate; CI = 95% confidence interval; ES = Cohen’s d effect size; * *p* < 0.05 significantly different from pre; # *p* < 0.01 significantly different from pre; ^∧^ *p* < = 0.001 group × time interaction.

## Supplementary Materials

The following are available online at https://www.mdpi.com/2411-5142/5/1/19/s1, Table S1: Lower Body Workout Example, Table S2: Upper Body Workout Example; Table S3: Raw Data Spreadsheet.
